# Development efficiency of green logistics in Guangdong province based on Super-SBM and GML index

**DOI:** 10.1371/journal.pone.0331893

**Published:** 2025-09-09

**Authors:** Yun Wang

**Affiliations:** School of Emergency Technology, Guangzhou Vocational College of Technology and Business, Guangzhou, China; Yunnan University, CHINA

## Abstract

Under China’s national sustainability strategy, the logistics industry is confronted with the imperative of high-quality green development. Given its status as the leading economic province and a national logistics hub, investigating green logistics development in Guangdong province holds paramount strategic importance. To comprehensively evaluate green logistics development efficiency of 21 cities in Guangdong from 2016 to 2022, this study employed the super-efficiency slacks-based measure model (Super-SBM) with undesirable outputs, the Global Malmquist–Luenberger (GML) productivity index and a four-quadrant analysis based on static and dynamic efficiency. With its integrated framework, this study dynamically assesses and meticulously classify green logistics efficiency in Guangdong, thereby offering unique perspectives and deep insights for regional policy formulation. The results reveal three key aspects. First, Guangdong’s overall green logistics efficiency remains relatively low with a slight downward trend, exhibiting significant regional and inter-city disparities. The Pearl River Delta(PRD) region, particularly Shenzhen and Guangzhou, consistently shows the highest and increasing efficiency, contrasting sharply with other regions. Second, technological progress is identified as the primary driver of changes in green logistics efficiency. Third, the four-quadrant analysis reveals distinct patterns among the 21 cities, classifying them into four categories: “high-efficiency-growth” (e.g., Shenzhen and Guangzhou), “high-efficiency-regression” (e.g., Dongguan), “low-efficiency-growth” (e.g., Zhuhai and Zhaoqing), and “low-efficiency-regression” (e.g., Meizhou and Shantou). Finally, this study puts forward key policy recommendations to promote green logistics development in Guangdong, including driving green transformation through the realignment of industrial and economic foundations, strengthening core cities’ green logistics leadership and regional collaboration, enhancing technological innovation capacity, and implementing precise and targeted support for cities across all four quadrants.

## 1. Introduction

Fueled by global economic expansion, the increasing scarcity of resources and the continuous degradation of environmental have solidified sustainable development as a worldwide consensus. As a fundamental pillar of the economy, the logistics industry plays a critical role in supporting overall economic growth and the facilitation of international trade [1]. However, it is also energy-intensive and a significant contributor to pollution, particularly in rapidly growing economies [2]. In recent years, carbon emissions from China’s logistics industry have accounted for 18% of the nation’s total carbon output and continue to grow annually [3]. According to the research report “Carbon Emissions of China’s Express Delivery Industry”, carbon emissions increased from 18.37 million tons in 2017 to 55.65 million tons in 2022 with a compound annual growth rate of nearly 25%. This rapid extensive growth model is unsustainable, making the green transformation of China’s logistics industry an imperative for the government’s sustainability agenda. A key approach to achieving this sustainable advancement is green logistics. Through the integration of environmental objectives into logistics planning and operational processes, green logistics aims to minimize the environmental impact of logistics activities while ensuring supply chain efficiency and reliability [4]. In current green logistics studies, green logistics efficiency, as a crucial measure of the development level and influencing factors of green logistics, has received widespread attention from the academic community and policymakers.

As one of China’s most economically dynamic provinces, Guangdong has ranked first in GDP nationwide for 36 years since 1989. Meanwhile, as a national logistics hub, its decades of economic expansion and logistical dominance have concurrently generated significant environmental burdens, making the pressure for energy saving and emission reduction particularly acute in the province. According to the World Resources Institute study, Guangdong had the highest total transport-related carbon emissions among all provinces from 2012 to 2020 [5]. Similarly, a recent nationwide study on the temporal and spatial evolution of logistics-related carbon emissions identified Guangdong as a top-ranking province in logistics-sector carbon output [6]. Given Guangdong’s economic importance and the significant environmental challenges confronting its logistics industry, systematic research on green logistics efficiency is urgently needed. While existing studies have explored various aspects of green logistics or regional logistics development, a comprehensive academic understanding of green logistics efficiency within Guangdong, particularly encompassing its entire geographical scope and all 21 cities, remains limited. Most existing studies tend to consider Guangdong as an aggregate within national datasets [1,5,6] or focuses on individual parts of Guangdong [3], lacking a comprehensive green efficiency analysis encompassing the entire province and its 21 cities.

Against this background, the primary objective of this study is to comprehensively evaluate both the static and dynamic green logistics efficiency of Guangdong and its 21 cities, and to identify the key factors influencing these efficiencies to inform targeted policy recommendations. To achieve this, this study employs the super-efficiency slacks-based measure (Super-SBM) model with undesirable outputs, combined with the Global Malmquist–Luenberger (GML) productivity index and a four-quadrant analysis framework, to evaluate both the static and dynamic green logistics efficiency of Guangdong and its 21 cities. Compared to previous studies, this study makes three key contributions. First, it advances the methodological toolkit by integrating the Super-SBM model, the GML index, and quadrant analysis to provide a comprehensive assessment of green logistics performance. Second, it offers an in-depth diagnosis of development levels, spatial patterns, and critical influencing factors, delivering robust empirical evidence to guide targeted policymaking for Guangdong. Third, given Guangdong’s economic prominence and representative nature, the findings serve as a valuable benchmark for other regions seeking to develop green logistics and formulate region-specific sustainability strategies.

## 2. Literature review

Traditional logistics performance is typically defined as the systematic evaluation of logistics activities in terms of cost-efficiency, operational effectiveness, and service levels, with the primary goal of ensuring the timely and economical movement of goods [7]. However, under the increasing pressures of resource scarcity and environmental degradation, this narrow focus has become inadequate as it fails to account for environmental impacts, such as carbon emissions, which are critical for sustainable development across industries [8,9]. To address this gap, Paul R. Murphy [10] first introduced the concept of green logistics, integrating ecological economics, sustainable development, and environmental ethics into logistics decision-making and operations. While there is no universally standardized definition, green logistics performance is widely understood as the capacity of a logistics system to achieve desirable economic outputs while minimizing resource inputs and environmental externalities, particularly carbon emissions. Based on these principles, green logistics efficiency can be defined as the capacity indicator of a logistics system to achieve optimal operational results (e.g., freight turnover) while minimizing environmental externalities (e.g., carbon emissions), under the constraint of available resource inputs (e.g., labor, capital, fuel). This efficiency-oriented framework offers a more holistic basis for evaluating logistics sustainability, aligning both operational performance and environmental responsibility.

The scientific assessment of green logistics efficiency is pivotal for achieving regional sustainable development. Early studies predominantly employed a data envelopment analysis (DEA) model to conduct pioneering evaluations of green logistics efficiency [11–13]. These studies often treated undesirable outputs like carbon emissions by incorporating them as inputs or through data transformation to maintain them as outputs. Wang and He [11] employed a DEA model to evaluate the environmental efficiency of regional transportation industries in China. Liu and Sun [12] employed DEA-based green total factor productivity (TFP) analysis to study China’s logistics industry. Chen et al. [13] utilized DEA to examine the spatiotemporal evolution of logistics efficiency under low-carbon constraints. These studies consistently revealed a pronounced macro-level feature: significant regional disparities in efficiency, with economically advanced eastern regions substantially outperforming other counterparts, where carbon abatement costs are notably higher. Nevertheless, they failed to adequately account for the drivers of regional efficiency disparities and lacked a dynamic perspective on efficiency changes.

Given traditional DEA models’ inherent limitations in dynamically tracking efficiency and explaining these underlying causes, many studies have integrated the DEA with other models, especially the Malmquist-Luenberger (ML) index for a more comprehensive analysis [6,14–18]. Yang et al. [6] used a DEA model and the ML index to evaluate logistics carbon emissions performance across 16 Chinese cities, finding significant regional disparities driven by economic development and resource allocation. Wang [16] proposed that the improvement of technological progress efficiency is the main factor to promote the continuous improvement of cost reduction and logistics efficiency in China by a DEA model and the ML index. Shen [17] used a DEA model and the ML index to assess the green logistics industry efficiency in the Beijing-Tianjin-Hebei region, finding that the average growth rate was 4.3%, with a volatile trend. Compared to earlier studies, these studies delved into a more granular analysis of efficiency, with the emergence of spatiotemporal evaluations for particular regions or cities. They consistently confirmed significant regional heterogeneity in green logistics efficiency within these scales, alongside a general upward trend in logistics efficiency over time [16,17]. Furthermore, several studies identify technological progress as a key driver of efficiency improvements [14,16].

Traditional DEA models have been criticized for their inability to effectively incorporate undesirable outputs into efficiency assessments and to differentiate and rank decision-making units (DMUs) deemed “efficient” with a score of 1 [18]. Additionally, the period-by-period calculation of the ML index limits its capacity to capture long-term efficiency trends due to a lack of intertemporal comparability [19]. These methodological limitations have collectively driven the development of advanced evaluation frameworks, notably the super-efficiency slack-based measure (Super-SBM) and the Global Malmquist-Luenberger (GML) index [20–23]. He et al. [20] investigated the green logistics efficiency of China’s three major bay city clusters by the Super-SBM model. Their findings indicated that transportation structure and urbanization negatively impacted green logistics efficiency, while technological innovation and logistics industry labor productivity had a positive influence. Additionally, efficiency trends in the three major city clusters varied, showing both increases and decreases. Meng and Qu [22] utilized the Super-SBM and the GML index to evaluate green logistics efficiency across 29 Chinese provinces, revealing significant provincial disparities and a gradual decline in overall efficiency, primarily due to energy overuse offsetting technological advancements. The findings of this study regarding a declining efficiency trend contradicts conclusions from earlier studies which suggested an upward trend in efficiency using DEA and the ML index. This discrepancy, in part, underscores the complexity and the sensitivity of various models to different specifications. Specifically, the Super-SBM model and the GML index are better equipped to accurately assess green logistics efficiency.

The aforementioned studies have substantiated key findings on regional efficiency disparities and the role of technological progress in enhancing efficiency with greater precision. Given significant discrepancies in efficiency assessments, more detailed regional analyses are crucial. These analyses, employing advanced methodologies, help truly identify the unique internal dynamics of key economic entities. As China’s largest economic province and a major contributor to carbon emissions, does Guangdong possess distinct patterns of efficiency evolution and unique internal influencing factors? Our understanding remains highly limited because previous research has either treated Guangdong as a single entity within national datasets or only focused on specific local regions, lacking systematic studies encompassing all 21 prefecture-level cities in the province. Therefore, employing the Super-SBM model and the GML index to conduct a spatiotemporal efficiency study of Guangdong Province’s 21 cities is not only necessary but crucial.

## 3. Methodology and data

### 3.1. Super-SBM model

The DEA framework is a non-parametric analytical approach based on the relative comparison of decision-making units (DMUs), and is well-suited for assessing the relative efficiency of complex systems with multiple inputs and outputs. Its core principle lies in constructing an efficiency frontier to evaluate the relative performance of each DMU. To allow for more accurate efficiency assessment, especially with undesirable outputs, Tone [24] proposed the SBM model, an extension of the DEA framework that incorporates slack variables. However, the standard SBM’s upper efficiency bound of 1 prevents further discrimination among efficient DMUs. This limitation was addressed by Tone’s [25] Super-SBM model, which allows efficiency scores to exceed 1, enabling comprehensive ranking of frontier units. The Super-SBM model relies on several key assumptions, including:

- Non-Parametric & Convex Technology: There is no specific functional form for the production technology, which is considered convex.
- Disposability of Inputs & Outputs: Inputs and desirable outputs are strongly disposable. Undesirable outputs are weakly disposable (costly to reduce) and jointly produced with desirable outputs.
- Efficiency Measurement: Efficiency is determined by maximizing output and minimizing input slacks. The super-efficiency extension allows for discrimination among efficient units by permitting scores greater than one.
- Returns to Scale: The analysis can be used under either Constant Returns to Scale (CRS) or Variable Returns to Scale (VRS). For this study, considering the varying development stages and operational scales of the analyze21 cities, the VRS assumption was adopted.

The specific model formulation is presented in Equation 1.


$$\text{min}\;\rho^{*}=\frac{1+\;\frac{1}{m}\sum_{i=1}^{m}\frac{S_{i}^{-}}{x_{ik}}}{1-\;\frac{1}{q_{1}+q_{2}}\left(\sum_{r=1}^{q_{1}}\frac{S_{r}^{+}}{y_{rk}}+\sum_{t=1}^{q_{2}}\frac{S_{t}^{b^{-}}}{b_{tk}}\right)}$$ (1)

$$s.t.\{\begin{array}{c} {}*20cx_{ik}\geq \sum_{j=1,j\neq k}^{n}x_{ij}\lambda_{j}-s_{i}^{-}\\ y_{rk}\leq \sum_{j=1,j\neq k}^{n}y_{rj}\lambda_{j}+S_{r}^{+}\\ b_{tk}\geq \sum_{j=1,j\neq k}^{n}b_{tj}\lambda_{j}{\;-\;S}_{t}^{b^{-}}\\ \sum_{j=1,j\neq k}^{n}\lambda_{j}=1\;\\ \lambda ,\;s^{-},s^{+}\geq 0\\ i=1,2,\cdots ,m;r=1,2,\cdots ,q_{1};\\ t=1,2,\ldots ,q_{2};j=1,2,\ldots ,n\left(j\neq k\right) \end{array}$$

In this Equation, *n*, *m*, *q*_1_, and *q*_2_ represent the total number of DMUs, input variables, desirable outputs, and undesirable outputs, respectively. The slack variables for the input variables, desirable outputs, and undesirable outputs are denoted by $s_{i}^{-}$, $S_{r}^{+}$, and $S_{t}^{b^{-}}$, respectively. Specifically, $x_{ik},\;y_{rk}$, and $b_{tk}$ represent the *i-th* input variable, *r-th* desirable output, and *t-th* undesirable output of the *k-th* DMU, respectively. $\lambda_{j}$ is the intensity vector for the *j-th* DMU in the reference set. Finally, $\rho^{*}$denotes the super-efficiency score of the *k-th* DMU.

### 3.2. GML index

While the Super-SBM model cannot capture temporal changes in the performance of assessment units, the Malmquist index was introduced into the dynamic productivity measurement framework [26]. However, it does not account for undesirable outputs. To overcome this, the ML index was developed by integrating the Directional Distance Function (DDF) with the traditional Malmquist index, thus incorporating undesirable outputs [27]. The ML index, by relying on a sequential (or contemporaneous) reference technology, suffers from issues such as non-circularity, limited comparability across periods, and infeasibility under certain linear programming conditions. To resolve these limitations, the GML index was developed by combining DDF with a global reference technology set. This global technology ensures consistent comparisons across all time periods. The GML index not only resolves infeasibility problems under variable returns to scale but also avoids technological regress caused by inward-shifting frontiers. The GML index’s assumptions regarding non-parametric and global convex technology, disposability of inputs and outputs, and variable returns to scale are consistent with those of the Super-SBM model. Simultaneously, the GML index introduces a critical assumption to facilitate inter-temporal comparison: Consistent Global Comparison. This means that by constructing a global production technology frontier spanning all time periods, it ensures consistent comparisons across time, satisfies the circularity property, and effectively avoids issues. The specific model is presented in Equation 2.


$$GML_{t}^{t+1}=\frac{1+{\vec{D}}^{G}\left(x^{t},y^{t},b^{t};y^{t},-b^{t}\right)}{1+{\vec{D}}^{G}\left(x^{t+1},y^{t+1},b^{t+1};y^{t+1},-b^{t+1}\right)}\;$$ (2)

In the Equation, $GML_{t}^{t+1}$ represents the total factor productivity change (TFP) from time *t* to *t* + 1, reflecting dynamic efficiency. ${\vec{D}}^{G}$ deno*t*es the global direc*t*ional distance function, where ${\;(y}^{t},-b^{t})$ or ${\;(y}^{t+1},-b^{t+1})$ serve as the directional vectors, indicating either an increase in desirable outputs or a decrease in undesirable output. *x*^*t*^, *y*^*t*^, and *b*^*t*^ are vectors of input variables, desirable outputs, and undesirable outputs, respectively. Likewise, *x*^*t*+1^, *y*^*t*+1^, and *b*^*t*+1^ represent these vectors at time t + 1. To further examine the underlying drivers of productivity change, this study adopts the decomposition approach proposed by Fare et al. [28], under the GML index framework. Total factor green productivity change is decomposed into generalized technical efficiency change (GEFFCH) and technological progress change(GTECH). Moreover, GEFFCH is further disaggregated into pure technical efficiency change (GPECH) and scale efficiency change (GSECH). This decomposition facilitates the identification of the principal contributors to productivity shifts. The specific model is presented in Equation 3.


         GML=GEFFCH*GTECH=GPECH*GSECH*GTECH   (3)

A GEFFCH value greater than 1 indicates that the decision-making unit has improved its use of production technology, resource allocation, and management efficiency compared to the previous period, reflecting an enhancement in technical efficiency. A GTECH value greater than 1 suggests that the outward expansion of the technological frontier has contributed to overall productivity growth. GPECH and GSECH indicators provide further insights into the sources of changes in technical efficiency. Specifically, GPECH > 1 signifies an improvement in resource utilization efficiency under given scale conditions, whereas GSECH > 1 implies a more optimal production scale and enhanced returns to scale. Conversely, values less than 1 for any of these indicators indicate regression in the corresponding dimension.

### 3.3. Four-quadrant analysis

To provide a more nuanced and comprehensive understanding of the diverse green logistics efficiency performance across Guangdong’s cities, this study employs a four-quadrant analysis framework. This analytical tool serves to graphically categorize and interpret the results obtained from the Super-SBM model and the GML index. This approach is particularly valuable given the well-established notion of significant regional efficiency disparities highlighted in prior research, which often remain obscured by aggregate analyses [11–16,21,29].

The framework is constructed by plotting cities based on two key dimensions: average green logistics static efficiency (X-axis) and full-period dynamic efficiency (Y-axis). The vertical threshold (Y-axis) is definitively set at 1, clearly distinguishing between cities with efficiency growth and decline. The horizontal threshold (X-axis) for the static efficiency score will be determined based on the empirical distribution of the cities’ average efficiency scores obtained from our analysis, ensuring a meaningful division between relatively high and low efficiency levels. Cities are then classified into four distinct quadrants based on their respective thresholds. Each quadrant represents a unique efficiency performance profile, enabling more targeted analysis and policy formulation.

### 3.4. Indicator system and data processing

#### 3.4.1. Indicator system.

Drawing upon established research in regional logistics efficiency evaluation [16,21–24], this study constructs a robust input-output evaluation framework for assessing the green logistics industry. Indicator selection was rigorously guided by principles of systematicness, scientific rigor, and feasibility. The complete indicator system is detailed in Table 1.

**Table 1:** Input and output evaluation indicator system.

Variable	Indicator	Explanation
Input variables	Fixed asset investment	Capital input
	Number of freight vehicles	Equipment input
	Number of employees at year-end	Labor input
Output variables	Freight turnover	Expected output – service volume
	Gross output value	Expected output – economic output
	Carbon emissions	Undesirable output – CO₂ emissions

Input indicators are essential for quantifying the resources utilized in logistics activities. Facilities such as warehouses, distribution centers, and specialized logistics infrastructure constitute fixed assets, representing long-term productive capital crucial for the efficient operation of the entire logistics network. Fixed asset investment, as a form of new capital formation, directly contributes to asset accumulation and serves as a primary determinant of output capacity and operational scale. Freight vehicles represent pivotal production assets for road transportation within logistics, with their numbers directly dictating road transport capacity. Given that road freight has consistently accounted for over 75% of Guangdong’s total freight volume since 2016 (Guangdong Comprehensive Transportation Statistical Yearbook), freight vehicles are included as a crucial stock asset indicator reflecting logistics service capacity. The Number of employees at year-end quantifies the labor input within the logistics industry. Characterized as a labor-intensive industry in China, the logistics necessitates substantial human resources for critical processes including cargo sorting, transportation, warehousing management, and last-mile delivery services. Number of employees at year-end reflects the overall scale of human capital.

Output indicators capture the outcomes generated by logistics activities, categorized into desirable economic and service outputs, and undesirable environmental outputs. Freight turnover quantifies the extent of logistics service provision by measuring the product of transported goods volume and distance. This indicator directly reflects the physical throughput and operational scale of logistics activities, capturing the core service function of physical distribution. The Gross output value is a monetary indicator representing the economic value generated by the logistics industry. It captures the industry’s financial contribution to regional economic activities and its overall market scale. The integration of freight turnover and gross output value offers a comprehensive assessment of the logistics industry’s dual role in facilitating physical trade and generating economic wealth. Carbon emissions, primarily stemming from fossil fuel combustion in transport vehicles and energy consumption in logistics facilities, serve as a direct and effective measure of the environmental burden imposed by logistics activities. Given that green logistics fundamentally emphasizes achieving economic benefits while minimizing environmental impact, carbon emissions are included as a vital undesirable output indicator.

#### 3.4.2. Data collection and processing.

As a composite industry, the logistics is not listed in China’s National Economy Industry Classification. Direct statistical data is not available in China. Since the transportation, warehousing, and postal industry occupy for approximately 80% of the overall logistics industry, lots of studies choose its data as a substitute for logistics [12,14,16,18]. Given the lack of city-level carbon emission data specific to the logistics sector, this study estimates logistics-related emissions indirectly. This method calculates logistics-related carbon emissions by first determining the share of the transportation, warehousing, and postal industry’s added value in a city’s total GDP. This share is then applied proportionally to the city’s total carbon emissions. This method, based on the assumption of a positive correlation between industrial carbon intensity and economic output, has been widely applied in prior studies on emission decomposition and industrial structure effects [30–32].

As fixed asset investment and Gross output value are nominal economic indicators measured in monetary terms, subject to price index fluctuations, their data were deflated to ensure inter-temporal comparability. Given that the fixed asset investment price index for Guangdong was discontinued from 2020 onwards, data for 2016–2019 were deflated using their respective annual Guangdong fixed asset investment price index. For the subsequent period of 2020–2022, the Industrial Producer Purchasing Price Index (IPPPI) was employed as a proxy. This combined approach can derive a consistent series of constant-price fixed asset investment figures for the entire 2016–2022 period. For the real gross output value, this study deflated its nominal figures using the gross domestic product (GDP) deflator calculated based on Guangdong’s nominal and constant-price GDP. Given that the base year for constant-price GDP shifted in 2020 (with 2015 serving as the base year for 2016–2020, and 2020 for 2021–2022), a base year conversion was implemented for the GDP deflator indices from 2020 onwards. This process unified all indices to a common 2015 base year, thereby ensuring the comparability of the entire time series.

Data in this study were mainly sourced from the 2017–2023 editions of the Guangdong Statistical Yearbook (published by the Guangdong Provincial Bureau of Statistics), municipal statistical yearbooks (published by respective municipal statistics bureaus), the China city statistical yearbook and China energy statistical yearbook (both published by the National Bureau of Statistics of China), and the China city carbon dioxide emission dataset within the CEADs (Carbon emission accounts and datasets) database. These sources collectively provide data covering the period 2016–2022.

## 4. Results and discussions

### 4.1. Static efficiency analysis of green logistics

This study employs MaxDEA software to assess static efficiency of green logistics for cities in Guangdong from 2016 to 2022 using the Super-SBM model. Consistent with established literature, the arithmetic mean method was adopted to calculate the static efficiency for each city over the entire study period [6,11,14,33]. Guangdong comprises 21 cities, which are categorized into four regions. These include the Pearl River Delta (PRD) region (Guangzhou, Shenzhen, Foshan, Dongguan, Zhongshan, Zhuhai, Jiangmen, Huizhou, and Zhaoqing), the East Wing region (Shantou, Jieyang, Chaozhou, and Shanwei), the West Wing region (Zhanjiang, Maoming, and Yangjiang), and the Mountain region Shaoguan, Qingyuan, Yunfu, Heyuan, and Meizhou). To better assess performance at the provincial and regional levels, the arithmetic mean method was also applied to calculate their respective green logistics efficiencies. The detailed data are presented in Table 2.

**Table 2:** Static green logistics efficiency of Guangdong (2016–2022).

Region	Year							Mean	Rank
	2016	2017	2018	2019	2020	2021	2022		
**Guangzhou**	0.8181	1.0530	0.9151	0.8681	1.0211	1.0015	1.0071	0.9548	2
**Shenzhen**	1.0285	1.0224	1.0044	1.0249	1.0005	1.1427	1.0275	1.0358	1
**Zhuhai**	0.0612	0.0364	0.0532	0.1010	0.1350	0.1735	0.1543	0.1021	18
**Shantou**	0.2094	0.1620	0.1532	0.1039	0.0483	0.0720	0.0729	0.1174	16
**Foshan**	0.2497	0.2435	0.3992	0.1714	0.1756	0.2576	0.2489	0.2494	7
**Shaoguan**	0.1521	0.1331	0.1349	0.1686	0.1273	0.1352	0.1668	0.1454	15
**Heyuan**	0.1055	0.0704	0.0673	0.0459	0.0309	0.0467	0.0720	0.0627	21
**Meizhou**	0.0988	0.0887	0.0861	0.0879	0.0487	0.0873	0.0799	0.0825	20
**Huizhou**	0.3277	0.2665	0.2357	0.2133	0.1449	0.1627	0.1521	0.2147	10
**Shanwei**	0.0789	1.0527	0.6627	0.1386	0.0472	0.0479	0.0423	0.2958	6
**Dongguan**	0.4473	1.0234	0.7342	0.3661	0.3861	0.4839	0.3918	0.5476	3
**Zhongshan**	0.3314	0.2763	0.2006	0.1021	0.0620	0.0668	0.0688	0.1583	14
**Jiangmen**	0.2148	0.2240	0.2012	0.1874	0.1558	0.1764	0.1660	0.1894	13
**Yangjiang**	0.3546	0.4118	0.2772	0.1759	0.1015	0.0931	0.0882	0.2146	11
**Zhanjiang**	0.3514	0.3798	0.3547	0.3265	0.1950	0.2549	0.2618	0.3034	5
**Maoming**	0.2306	0.2079	0.2027	0.2509	0.1965	0.2668	0.2344	0.2271	8
**Zhaoqing**	0.0729	0.0713	0.0740	0.0766	0.0742	0.1040	0.1067	0.0828	19
**Qingyuan**	0.2971	0.3006	0.2957	0.1531	0.0989	0.1203	0.2557	0.2173	9
**Chaozhou**	0.2852	0.3069	0.3999	1.0540	0.1913	0.1810	0.2098	0.3754	4
**Jieyang**	0.1524	0.1691	0.1062	0.1127	0.0552	0.0520	0.0784	0.1037	17
**Yunfu**	0.1643	0.2873	0.1506	0.1276	0.1516	0.2551	0.2589	0.1993	12
**Province Total**	0.2872	0.3708	0.3195	0.2789	0.2118	0.2465	0.2449	0.2800	--
**Pearl River Delta(PRD)**	0.3946	0.4685	0.4242	0.3457	0.3506	0.3966	0.3692	0.3928	1
**Eastern Wing**	0.1815	0.4227	0.3305	0.3523	0.0855	0.0882	0.1009	0.2231	3
**Western Wing**	0.3122	0.3332	0.2782	0.2511	0.1643	0.2037	0.1945	0.2481	2
**Mountain region**	0.1636	0.1760	0.1469	0.1166	0.0915	0.1287	0.1668	0.1414	4

The average static efficiency of green logistics in Guangdong from 2016 to 2022 is 0.2800, indicating a generally low efficiency level and substantial room for improvement. Regionally, PRD, the most economically advanced region, recorded the highest average efficiency at 0.3928. This high efficiency aligns with the PRD’s robust economic development and mature logistics infrastructure. In contrast, the mountainous region had the lowest average efficiency at just 0.1414, revealing pronounced spatial disparities in green logistics development. Such a stark contrast underscores the deep-seated economic imbalance across the province, where more developed regions demonstrate higher efficiency while less developed regions lag significantly.

At the city level, the top three performers in terms of average efficiency were Shenzhen (1.0358), Guangzhou (0.9548), and Dongguan (0.5476). These cities, serving as the leading economic drivers of Guangdong, naturally exhibit stronger green logistics capabilities. All other cities had average efficiency values below 0.5, with Heyuan ranking the lowest at only 0.0627. Overall, green logistics efficiency across Guangdong’s cities exhibits a polarized structure characterized by “high–low extremity”. A few leading cities perform well above average, while lagging cities remain far behind. Most medium-performing cities fall into the low-efficiency range, collectively yet to form a strong support base for the province’s high-quality green logistics development.

The overall green logistics efficiency in Guangdong was found to be at a relatively low level, a finding that diverges from some existing literature which suggests Guangdong’s green logistics efficiency is at the forefront [10,11,22]. This discrepancy primarily stems from a difference in the constructed production frontier employed. Previous studies often used a national or broader regional frontier, against which Guangdong performed exceptionally well. In contrast, this study evaluates Guangdong’s cities against a localized, within-province frontier. This also indirectly suggests that Guangdong’s most outstanding cities are also prominent nationwide. Consequently, when benchmarked against this stringent localized, within-province production frontier, the overall average efficiency of Guangdong appears comparatively lower. This finding indicates that while a few cities within Guangdong exhibited highly competitive efficiency levels, potentially among the highest nationwide, substantial intra-provincial disparities reveal significant regional imbalances in development. Therefore, Guangdong’s perceived overall efficiency advantage over other provinces may have been largely driven by the outstanding performance of a few individual cities.

### 4.2. Dynamic efficiency analysis of green logistics

#### 4.2.1. Overall dynamic efficiency analysis of green logistics.

This study employs MaxDEA software to assess the temporal evolution of green logistics efficiency for cities in Guangdong from 2016 to 2022 using the GML index. To ensure consistency with the static efficiency analysis, city-level GML indices were aggregated using the arithmetic mean to calculate the GML indices for each region and for Guangdong. Given the GML index’s multiplicative nature, a sequential multiplication method was further applied to derive the cumulative index for each city, region, and the province over the full period (2016–2022), thereby reflecting their long-term green logistics efficiency trajectories. The detailed data are presented in Table 3.

**Table 3:** Dynamic green logistics efficiency of Guangdong (2016–2022).

GML Index	Year						Full period GML index	Rank
	2016- 2017	2017- 2018	2018- 2019	2019- 2020	2020- 2021	2021- 2022		
**Guangzhou**	1.2173	0.8712	0.9786	1.0755	0.9956	1.0259	1.1401	5
**Shenzhen**	0.9665	0.9422	1.0505	0.7417	1.8486	0.9671	1.2685	3
**Zhuhai**	0.5828	1.3143	1.6002	1.1066	1.2864	1.0001	1.7451	1
**Shantou**	0.7273	0.8307	0.6216	0.6683	1.4644	1.1177	0.4108	19
**Foshan**	1.0711	1.4651	0.3024	1.1931	1.4154	0.9691	0.7766	13
**Shaoguan**	0.8697	1.0162	1.2393	0.8058	1.1437	1.1724	1.1835	4
**Heyuan**	0.6904	0.9716	0.6889	0.6914	1.4818	1.4936	0.7072	16
**Meizhou**	0.8839	1.0019	0.9885	0.5870	1.6593	0.9368	0.7987	11
**Huizhou**	0.8167	0.9116	0.9296	0.6913	1.1542	0.9188	0.5074	17
**Shanwei**	0.9145	0.8975	0.6222	0.7549	0.9181	1.0124	0.3583	21
**Dongguan**	1.8018	0.7786	0.5599	1.1012	1.1596	0.9140	0.9168	9
**Zhongshan**	0.8695	0.7994	0.7059	0.7131	1.0827	1.0710	0.4058	20
**Jiangmen**	0.9853	0.9544	0.9461	0.9403	1.0717	1.0053	0.9013	10
**Yangjiang**	1.5671	0.7611	0.4165	0.9143	0.8795	1.0334	0.4127	18
**Zhanjiang**	1.0323	1.0093	0.9142	0.6358	1.1969	1.0213	0.7402	14
**Maoming**	0.8774	0.9719	1.2832	0.8458	1.2787	0.9050	1.0710	6
**Zhaoqing**	1.0735	1.1850	0.7850	1.1897	1.1702	1.0747	1.4941	2
**Qingyuan**	1.0034	0.9753	0.4891	0.7186	1.2071	1.7187	0.7136	15
**Chaozhou**	1.0550	1.1873	1.1712	0.7427	0.8450	1.1467	1.0557	7
**Jieyang**	1.1109	0.8281	1.0470	0.9698	0.9755	1.1371	1.0362	8
**Yunfu**	1.2359	0.7217	0.7378	1.0627	1.1063	1.0272	0.7947	12
**Province Total**	1.0168	0.9712	0.8608	0.8643	1.2067	1.0794	0.9570	--
**Pearl River Delta(PRD)**	1.0427	1.0247	0.8731	0.9725	1.2427	0.9940	1.1207	1
**Eastern Wing**	0.9519	0.9359	0.8655	0.7839	1.0507	1.1035	0.7009	4
**Western Wing**	1.1589	0.9141	0.8713	0.7986	1.1184	0.9865	0.8133	3
**Mountain region**	0.9367	0.9373	0.8287	0.7731	1.3196	1.2697	0.9426	2

Green logistics efficiency in Guangdong exhibited a fluctuating trend with a slight overall decline based on the GML index value of 0.9570 during the 2016–2022 period. Specifically, it showed a slight increase from 2016 to 2017, a continuous decline from 2017 to 2020, and then a slight increase again from 2020 to 2022.

The increase of 2016–2017 may be attributed to the early positive effects of green development concept and policies outlined in China’s 13th Five-Year Plan and 13th Five-Year Plan for Energy Conservation and Environmental Protection in Transportation. According to statistics from the China Logistics Information Center, logistics efficiency has maintained a steady improvement during the 13th Five-Year Plan period.

However, a continuous decline was observed from 2017 to 2020. In 2016, China State Council issued the “Outline of the National Innovation-Driven Development Strategy”, which set forth a comprehensive deployment for accelerating the implementation of a national innovation-driven development strategy. As China’s leading economic province, Guangdong actively responded by initiating significant economic restructuring aimed at transforming from a “major manufacturing province” to a “manufacturing powerhouse”, and a heightened emphasis on ecological protection through stricter environmental regulations. However, such deep-seated economic restructuring inevitably introduces a period of “transitional growing pains” for the logistics industry. The relocation or upgrading of a significant volume of low-end manufacturing disrupted existing logistics networks and supply chains, while the burgeoning high-end manufacturing and knowledge-intensive service industries presented new, often more complex and demanding logistics requirements. Concurrently, the COVID-19 pandemic emerged in 2020, profoundly impacting the efficiency of various industries, including agriculture, manufacturing, and logistics as documented by several studies [34–36]. The superposition of these factors likely contributed to the observed decline in Guangdong’s green logistics efficiency during from 2017 to 2020.

Conversely, from 2020 to 2022, efficiency showed an upward trend. This improvement was likely facilitated by the cumulative benefits of green policies, the gradual progression of relevant technologies, and the post-pandemic economic recovery. Moreover, enhanced organizational resilience in responding to pandemic challenges may have also contributed to this renewed efficiency growth.

At the city level, there are 8 out of 21 cities experienced an increase in efficiency. Zhuhai exhibited the highest GML index, whereas Shanwei demonstrated the lowest. Four of the top five cities in the GML index are from PRD region, underscoring their strong performance and leadership. Conversely, cities like Shanwei and Shantou recorded GML indices below 1, highlighting their ongoing difficulties in achieving green logistics advancement. This divergence in dynamic efficiency broadly aligns with the spatial disparities observed in our static efficiency analysis, where PRD region consistently demonstrated higher efficiency, further underscoring the uneven development landscape across Guangdong.

#### 4.2.2. Decomposition analysis of dynamic efficiency.

**Fig 1:**
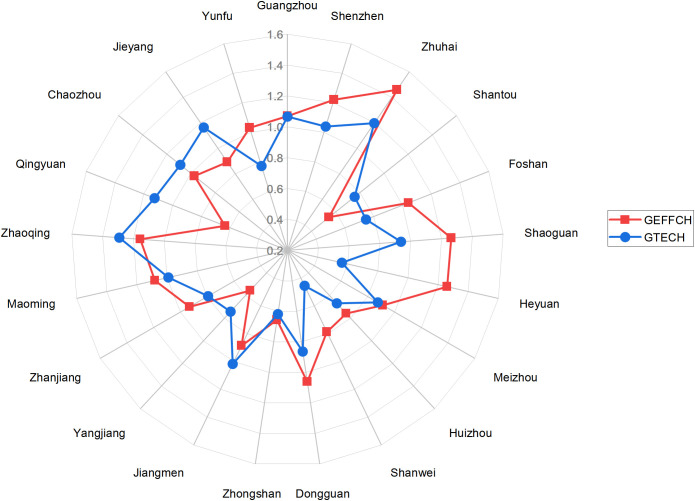
GEFFCH, GTECH indices of each city (2016-2022).

**Fig 2:**
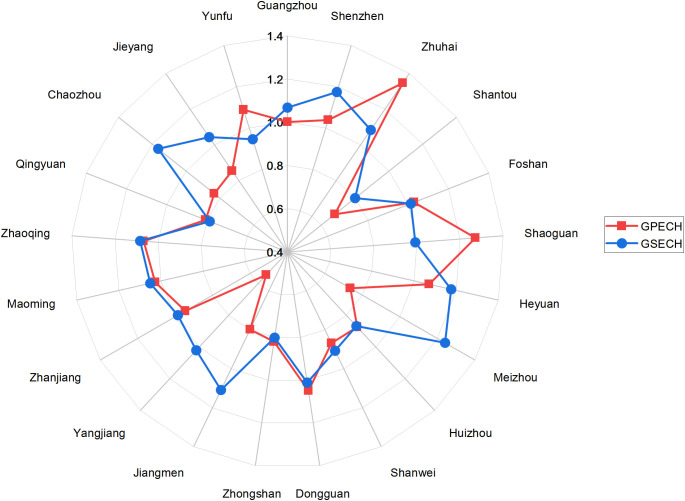
GPECH, GSECH indices for each city (2016-2022).

**Fig 3:**
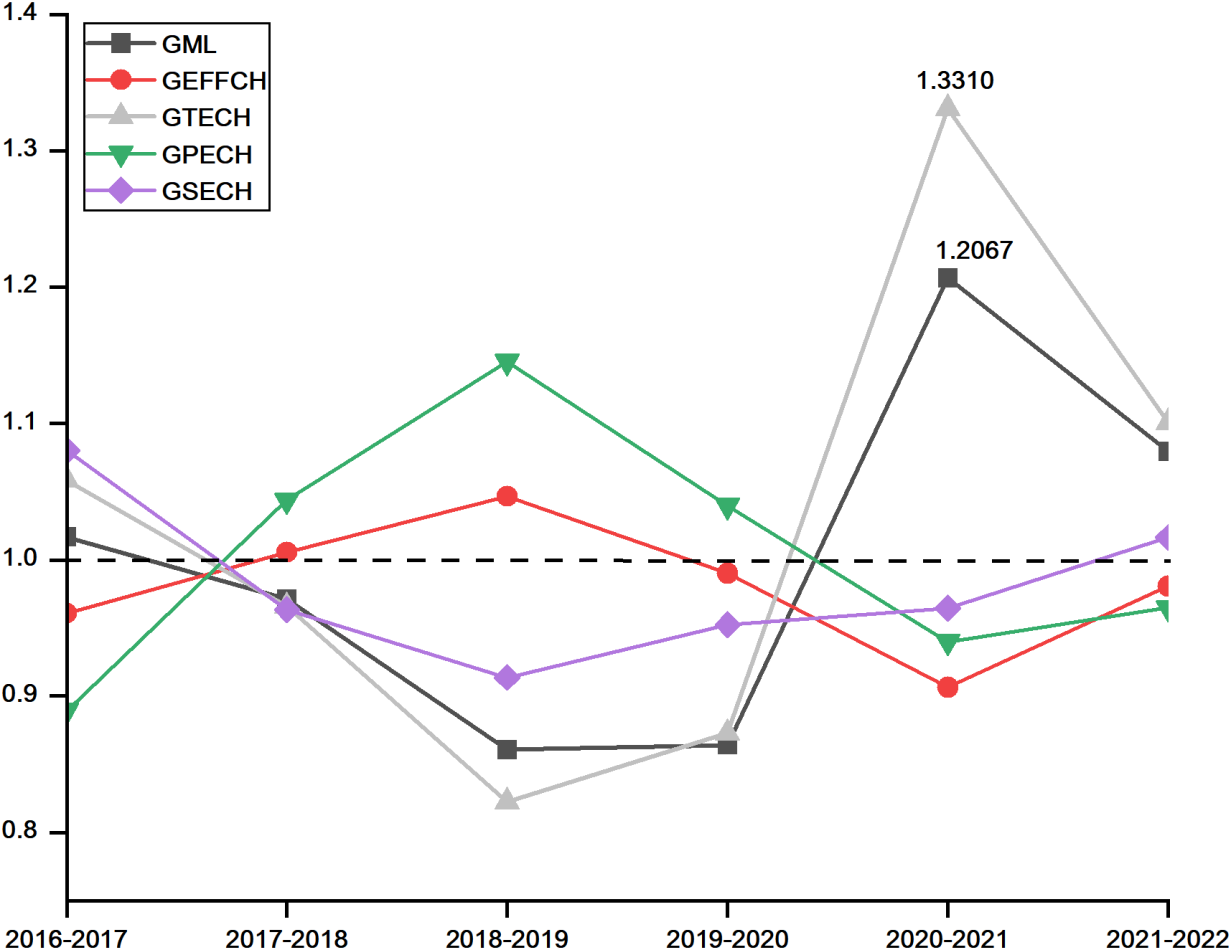
Trends of GML and its decomposition indices in Guangdong (2016-2022).

To further investigate the underlying mechanisms driving changes in green logistics efficiency and identify potential improvement pathways, this study employed a multiplicative approach to calculate full-period cumulative decomposition indices based on the GML index results for each city. These indices include GEFFCH, GTECH, GPECH and GSECH, as illustrated in Figs 1 and 2. Additionally, to provide a comprehensive understanding of the internal structure of overall efficiency changes at the provincial level, this study decomposes Guangdong’s GML index and visualizes the annual trends of its components in Fig 3. All decomposition indices are derived using the same methodology as the provincial GML index to ensure consistency and comparability across city-level, regional, and provincial data.

As depicted in Fig 3, the overall trends of the GML and GTECH indices exhibit a high degree of consistency across the years. Further analysis reveals a Pearson correlation coefficient of 0.6990 between these two indices, suggesting that green technological change as the primary underlying factor influencing green logistics efficiency. This suggests that Guangdong’s ability to effectively absorb and apply advanced green technologies significantly influences the periodic shifts in its green logistics performance.

This influence of technology is particularly evident during 2020−2021 period, in which GML and GTECH demonstrated a significant rebound, with each index reaching its peak. This advancement in the logistics industry’s technological frontier was collectively propelled by a series of pivotal policy interventions. Key policies included the Chinese government’s “Guiding Opinions on Accelerating the Establishment of a Sound Green, Low-Carbon, and Circular Development Economic System” and “Notice on Improving Financial Subsidy Policies for the Promotion and Application of New Energy Vehicles (2020)”. At the provincial level, supporting policies such as the “Guangdong Hydrogen Energy Industry Development Plan/Policies”, “Guangdong Carbon Peak Implementation Plan”, and “Guangdong Comprehensive Transport System 14th Five-Year Plan” (2020) collectively provided clear long-term strategic directions, financial subsidies, investment attraction, and robust support for infrastructure development specifically aimed at fostering green technological innovation and adoption. The integration of green and intelligent technologies into Guangdong’s logistics system was actively facilitated by these policy thrusts, especially during the critical COVID-19 control phase in 2021. Leading cities like Guangzhou and Shenzhen spearheaded the large-scale deployment of new energy logistics vehicles and unmanned delivery systems for essential services such as nucleic acid sample transfers, medical supply distribution, and contactless deliveries in quarantined areas. This deployment significantly reduced manual labor demands and mitigated cross-infection risks. Concurrently, the rapid adoption of intelligent warehousing systems, automated picking technologies, and advanced cold-chain storage and transportation solutions enabled highly efficient and environmentally sustainable emergency logistics operations for epidemic-related supplies. The high-frequency and broad-scale application of these green technologies effectively expanded the technological frontier of the logistics industry, which directly contributed to the spike in GML index to its cycle peak in 2020–2021.

However, GML index subsequently declined to 1.0657 in 2021–2022, reflecting a slowdown in the pace of technological progress. This deceleration may be attributed to the diminishing marginal returns observed from the initial surge in green technologies, coupled with ongoing fluctuations in the external economic environment and persistent supply chain disruptions during the later stages of the pandemic.

Furthermore, GEFFCH generally remained below 1 throughout most of the study period, particularly from 2019–2022, where it consistently acted as a hindering factor. This persistent low performance was likely due to pandemic-induced disruptions that undermined transport coordination and overall system efficiency. A further breakdown revealed that both GPECH and GSECH largely declined or remained below unity over this period, indicating that neither improvements in management efficiency nor the realization of optimal economies of scale significantly contributed to overall efficiency gains, and often served as a drag.

At the city level, as illustrated in Figs 1 and 2, a cluster of cities demonstrated robust performance in green logistics efficiency, consistently exhibiting both GEFFCH and GTECH values exceeding 1 throughout the study period. This strong performance is primarily driven by their advanced logistics infrastructure, robust policy support, and solid technological foundation. Specifically, Shenzhen and Guangzhou, as core cities of Guangdong, serve as crucial innovation and policy hubs. Zhuhai effectively leverages its unique position within the Hengqin Guangdong-Macao Deep Cooperation Zone to actively develop advanced cross-border and bonded logistics systems. Zhaoqing benefits from significant logistics agglomeration effects spurred by emerging e-commerce enterprises (Shein), propelling the optimization and green transformation of its logistics system. Furthermore, Chaozhou, representing the East Wing region, functions as a key regional center. These collective advantages enable them to achieve superior technical efficiency and technological progress in green logistics advancement. By contrast, cities such as Yunfu, Meizhou, and Shanwei exhibit lower GEFFCH and GTECH values, suggesting persistent limitations in both technical capacity and resource allocation efficiency. Further decomposition of GEFFCH reveals that Shanwei, Yangjiang, and Meizhou underperform in both GPECH and GSECH, indicating a dual bottleneck in their green logistics efficiency arising from weak management effectiveness and limited capacity for resource integration. Overall, our results reveal pronounced heterogeneity in Guangdong’s urban green logistics development, underscoring the need for localized, targeted policy interventions based on each city’s unique foundation and efficiency constraint.

### 4.3. Four-quadrant analysis: A static–dynamic perspective

**Fig 4:**
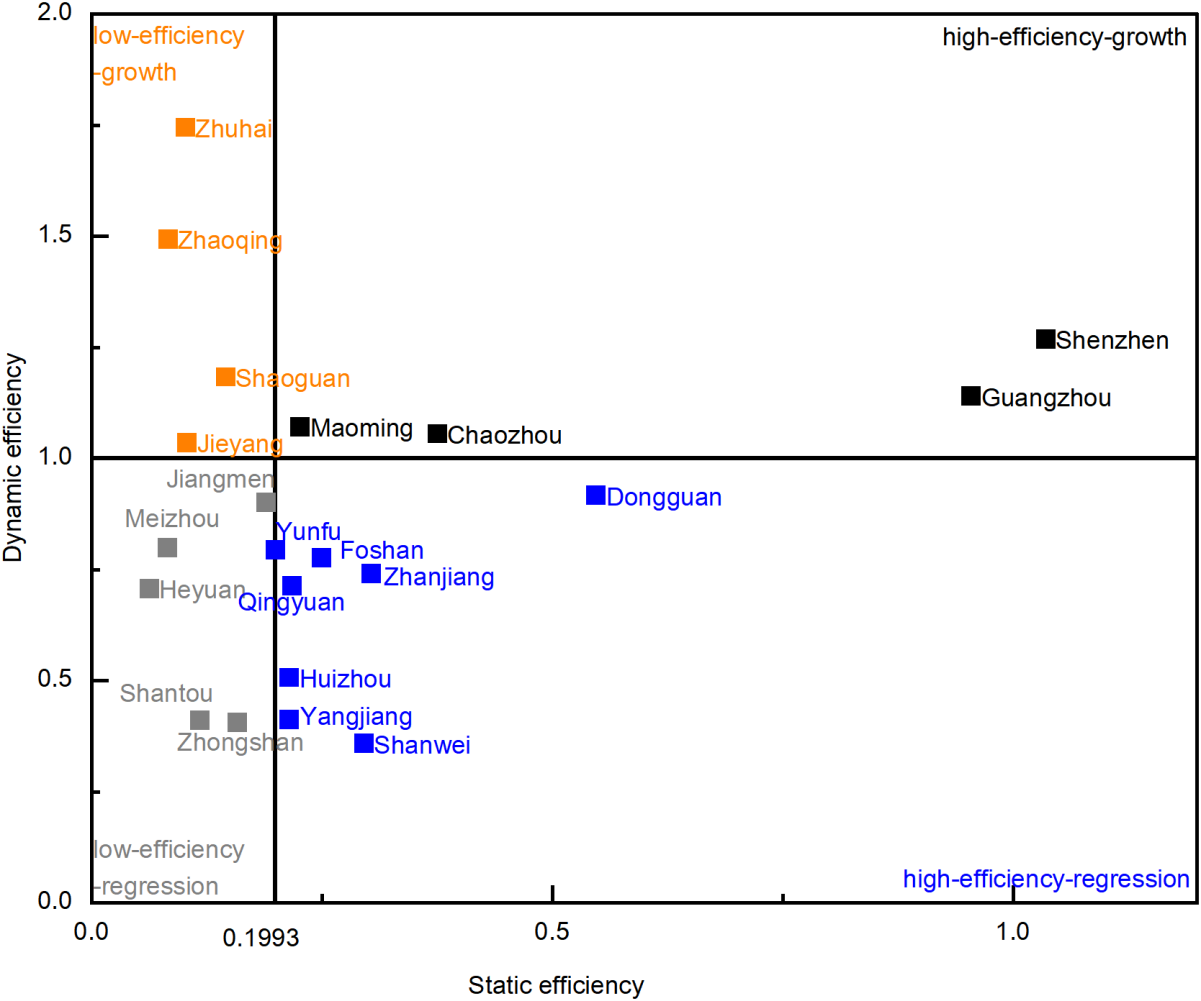
Four-quadrant analysis of green logistics efficiency in Guangdong (2016-2022).

Building upon the methodology of relevant studies [32,37] and utilizing the measured static and dynamic efficiency results, the distribution of the 21 prefecture-level cities in Guangdong is presented within a four-quadrant analytical framework, as shown in Fig 4. For the static efficiency division, the median Super-SBM value of 0.1993 was adopted as the horizontal threshold. This decision was grounded in the observed data distribution that static efficiency exhibited a significant right skew, with the presence of two extremely high values. Employing the median effectively mitigates the distortion of the dividing line’s position by these outliers, thereby leading to a more robust and representative categorization across efficiency levels. Furthermore, a sensitivity analysis confirmed the stability and appropriateness of this median-based dividing line. For the dynamic efficiency division, the GML index value of 1 was adopted as presented in the methodology. All cities were categorized into four quadrants: high-efficiency-growth, low-efficiency-growth, high-efficiency-regression, and low-efficiency-regression.

The high-efficiency-growth quadrant comprises four cities which are generally characterized by well-developed infrastructure, advantageous geographical locations, and robust policy support for green logistics development. Specifically, Shenzhen and Guangzhou serve as key engines driving the province’s high-quality green logistics due to their exemplary progress in smart logistics systems and the deep integration of next-generation information technologies into the industry. These factors jointly provide robust technological and market foundations for the advancement of green logistics.

The high-growth-regression quadrant includes eight cities, most of which were economically well-developed previously. However, following the economic transformation initiated in 2015, their subsequent adjustments proved largely unsatisfactory. Dongguan is the most representative city in this group. Historically, Dongguan served as a major global manufacturing base, with its logistics system supporting extensive export processing and goods distribution activities. However, amid global economic restructuring and domestic industrial upgrading, manufacture industry has increasingly shifted out of Dongguan in recent years. Traditional industries have lagged in transformation, resulting in significant changes in logistics demand. Meanwhile, despite the government’s promotion of green logistics policies, corresponding industrial guidance and structural adjustments have not kept pace, leading to a lack of new growth drivers for the green logistics industry. The efficiency challenges in green logistics are not solely attributable to internal operational issues, but also significantly influenced by broader external economic and industrial restructuring factors.

The low-efficiency-growth quadrant comprises four cities in which Zhuhai and Zhaoqing are representative examples. These cities have shown strong upward trends despite lower initial efficiency. As highlighted in our dynamic efficiency analysis, Zhuhai’s development is driven by its unique position within the Hengqin Guangdong-Macao Deep Cooperation Zone, while Zhaoqing’s growth stems from significant logistics agglomeration effects spurred by emerging e-commerce enterprises like Shein. The remaining two cities in this quadrant also exhibit similar positive trends in industrial transformation, contributing to their robust growth momentum.

The low-efficiency-regression quadrant encompasses five cities which are primarily characterized by a weak economic foundation. Meizhou and Shantou serve as representative examples from this group. Their industrial structures typically consist of traditional, resource-intensive, or low-value-added industries like conventional manufacturing and agricultural processing. These industries are prone to high environmental impact, provide minimal drivers for sophisticated logistics, and show limited progress in green transformation. Furthermore, undeveloped economy often translates into limited investment in advanced green technologies, modern infrastructure, and research and development for the logistics sector. These regions may also suffer from talent drain, as skilled labor and management personnel migrate to more developed areas. This outflow directly hinders innovation and the adoption of modern management practices crucial for green logistics advancement, exacerbating their decline.

Overall, four-quadrant analysis reveals pronounced inter-city heterogeneity in Guangdong’s green logistics development, underscoring the necessity of differentiated and adaptive strategies. Through analysis, green logistics performance is intricately linked to underlying economic foundations, industrial structure, and the effectiveness of policy transmission. The high-efficiency-growth quadrant exemplifies regions where robust economic bases, advanced infrastructure, and strategic policy alignment synergistically drive high-quality green logistics. Both the high-growth-regression and low-efficiency-regression quadrants, collectively comprising thirteen cities, face acute challenges primarily rooted in industrial and economic transitions. Their cases demonstrate that green logistics is profoundly shaped by broader external economic and industrial factors, not just internal sector operations. Cases from low-efficiency-growth quadrant show how effective policy and strategic industrial pivoting can drive green logistics advancement. Guangdong’s green logistics landscape is diverse and complex. Therefore, understanding each city’s unique position and its underlying economic and industrial drivers is crucial for formulating effective, localized, and sustainable province-wide policy interventions.

### 4.4. Policy implications

Based on the preceding results and discussions, the development of green logistics in Guangdong can be advanced by focusing on the following aspect:

#### 4.4.1. Drive green transformation through the realignment of industrial and economic foundations for logistics demand.

This strategy focuses on creating the essential industrial and economic conditions that inherently drive demand for green logistics, rather than just treating symptoms. This involves actively guiding traditional industries towards green, high-value production and attracting new green manufacturing and service sectors, which will fundamentally reshape the quality and type of logistics demand. For cities with weaker economies, prioritize foundational economic development that explicitly integrates environmental sustainability goals. Create the necessary financial and market impetus for investment in green logistics infrastructure and technologies, fostering cross-regional synergy that directly stimulates demand for green logistics services through broader economic initiatives.

#### 4.4.2. Strengthen core cities’ green logistics leadership and regional collaboration.

High-efficiency-growth cities, especially Shenzhen and Guangzhou should further leverage their leading and demonstrative roles in green logistics development. This involves continuously promoting technological innovation, disseminating effective management practices, and scaling system models to accelerate the green transformation of the PRD region. Concurrently, establishing cross-regional cooperation mechanisms for green logistics is crucial. This will achieve integrated advancements in policy alignment, technology sharing, and infrastructure interconnectivity, thereby fostering a province-wide green logistics system characterized by coordinated development and gradient synergy.

#### 4.4.3. Enhance technological innovation capacity.

Given the pivotal role of technological progress in improving green logistics efficiency, sustained support should be directed toward key technologies. Efforts should focus on breakthroughs in core areas such as green transportation equipment, intelligent warehousing systems, and recyclable packaging materials, with an emphasis on promoting the industrialization and large-scale adoption of technological achievements. Simultaneously, the collaborative innovation mechanism integrating government, industry, academia, research, and application should be improved to foster deeper integration among universities, research institutions, and leading logistics enterprises. This will enhance the independent innovation capacity of green logistics, strengthen talent cultivation and knowledge transfer, and provide continuous momentum and intellectual support for system upgrades.

#### 4.4.4. Implement precise and targeted support for cities across all quadrants.

Guangdong’s 21 cities exhibit diverse green logistics characteristics across four quadrants, each requiring tailored policy interventions.

- For high-efficiency-growth cities: The policy focus should be on consolidating and expanding their leading advantages. This includes continuous investment in cutting-edge green logistics technologies, optimizing resource allocation to support high-value green industries, and encouraging them to explore higher green standards and international collaborations to maintain their demonstrative role.
- For high-efficiency-regression cities: The primary challenge for these cities stems from lagging industrial transformation impacting demand. Policy should aim to reignite their green logistics development by addressing the industrial demand gap. Specific measures include: incentivizing the recalibration of existing industrial structures towards green and high-value production, proactively cultivating new industries whose inherent operational models generate sophisticated green logistics demand, and supporting the development of innovative green logistics solutions that directly facilitate this industrial transition and create new green growth drivers.
- For low-efficiency-growth cities: The policy core should be to accelerate their green logistics infrastructure and capability building. This involves prioritizing investment in green logistics infrastructure, providing comprehensive policy support, and strengthening their capacity for technology absorption and transfer, ensuring rapid growth translates into simultaneous efficiency improvements.
- For low-efficiency-regression cities: These cities face a dual challenge of weak economic foundations and traditional industrial structures. Policy must focus on fundamental structural improvement. This includes: prioritizing the enhancement of basic logistics infrastructure, increasing support for the green transformation of traditional industries, and utilizing regional cooperation mechanisms to gradually narrow the development gap and fundamentally improve their green logistics development capacity.

## 5. Conclusions

### 5.1. Main findings

This study comprehensively investigated the green logistics efficiency of 21 cities in Guangdong Province from 2016 to 2022, employing static, dynamic, and integrated perspectives. Our findings reveal Guangdong’s overall green logistics efficiency remains low, exhibiting a fluctuating yet slightly downward trend and significant developmental imbalance. The PRD region consistently demonstrated the highest and continuously improving efficiency, with Shenzhen and Guangzhou notably outperforming other cities. Marked regional and inter-city disparities were evident, intricately linked to underlying economic foundations, industrial structure, and policy effectiveness. While green technological progress served as a core driver of efficiency change during the period, overall green efficiency change remained unsatisfactory. These results collectively underscore the urgent need for tailored interventions to foster balanced green logistics development.

### 5.2. Contributions

Through a multi-perspective analytical framework, this study offers a more nuanced understanding of green logistics development in Guangdong. Contrary to conclusions in existing literature asserting high green logistics efficiency in Guangdong, this study reveals its substantial potential for enhancement and provides specific policy directions for its development. This can also serve as a reference model for green logistics efficiency development in other provinces.

### 5.3. Limitations and future research

This study also has several limitations. Due to the lack of a dedicated statistical classification for the logistics industry, data were proxied using figures from transportation, storage, and postal industries. The logistics industry’s urban carbon emissions data are also estimated. These data might introduce biases into the results. Therefore, future research will focus on exploring and constructing a more precise proxy indicator system, and applying advanced econometric methods to improve data accuracy, thereby enhancing the robustness and reliability of model results. Furthermore, in interpreting the findings, it is essential to fully acknowledge the significant inherent disparities among the 21 cities in Guangdong concerning economic development levels, geographical conditions, and policy environments. These complex heterogeneous factors likely exert intricate and profound influences on each city’s green logistics efficiency. Despite concerted efforts to capture this regional heterogeneity, the current analytical results may still not fully reflect all subtle nuances. Accordingly, future investigations will aim to employ more refined regional typologies or in-depth case study methodologies to comprehensively and meticulously explore the driving factors and development pathways of green logistics efficiency across diverse city types.
